# Performance of novel digital real-time PCR for detection of SARS-CoV-2, respiratory syncytial viruses, and influenza viruses in Ghana

**DOI:** 10.1128/spectrum.03219-24

**Published:** 2025-08-12

**Authors:** Michael Owusu, Bernard Nkrumah, Godfred Acheampong, Stephen Opoku Afriyie, Ebenezer Kojo Addae, Richard Larbi, Richard Owusu Ansah, Chrysantus Kubio, Farouk Saeed, Nana Kwame Ayisi-Boateng, Eric Darko, Jacob Amonoo-Neizer, Abena Gyawu Owusu-Ansah, Frederick Ayensu, Peter Kojo Brenya, Veronica Bannor, Pawan Angra, Danielle Thompson Barradas

**Affiliations:** 1 Centre for Health System Strengthening, Kumasi, Ghana; 2 Department of Medical Diagnostics, Kwame Nkrumah University of Science and Technology 98763, Kumasi, Ghana; 3 Genomic and Infectious Disease Laboratory, Kwame Nkrumah University of Science and Technology 98763, Kumasi, Ghana; 4 African Field Epidemiology Network, Accra, Ghana; 5 Regional Health Directorate, Ghana Health Service 285256, Savannah, Ghana; 6 University Health Services, Kwame Nkrumah University of Science and Technology 98763, Kumasi, Ghana; 7 Department of Medicine, Kwame Nkrumah University of Science and Technology 98763, Kumasi, Ghana; 8 HopeXchange Medical Centre, Kumasi, Ghana; 9 Asokwa Children’s Hospital, Kumasi, Ghana; 10 Division of Global Health Protection, Global Health Center, US Centers for Disease Control and Prevention 144823, Atlanta, Georgia, USA; City of Hope Department of Pathology, Duarte, California, USA

**Keywords:** respiratory viruses, SARS-CoV-2, RSV, influenza, Lab On An Array, RT-PCR, Ghana

## Abstract

Digital PCR (dPCR) systems offer high sensitivity and reproducibility without requiring external control standards. However, their performance against real-time reverse transcription-PCR (rRT-PCR) for detecting respiratory viruses remains unexplored in Ghana. We therefore evaluated the performance of a novel dPCR, Lab-On-An-Array (LOAA), for the detection of severe acute respiratory syndrome coronavirus 2 (SARS-CoV-2), respiratory syncytial virus (RSV), and influenza viruses type A (Flu A) and B (Flu B). A cross-sectional hospital-based study was conducted between August 2022 and January 2023 in Ghana’s Ashanti and Savannah Regions. Oropharyngeal swabs from 356 participants with a median age of 19 years, presenting with suspected respiratory illness, were tested using LOAA and rRT-PCR. Viral RNA was extracted using a Qiagen Viral Mini Kit (Qiagen Diagnostics GmbH, Germany). LOAA and rRT-PCR tests were performed using Genoplexor COVID-19/Flu/RSV Detection Kit (Optolane Technologies Inc, South Korea) and FluoroType SARS-CoV-2/Flu/RSV kits (Hain Lifescience GmbH, Germany), respectively. LOAA’s performance metrics were assessed using rRT-PCR as the gold standard. Overall positivity rates were 29.78% and 30.90% for LOAA and rRT-PCR, respectively. Compared to rRT-PCR, LOAA’s sensitivity was 87.76% for RSV, 91.30% for SARS-CoV-2, 86.21% for Flu B, and 88.89% for Flu A. Positive predictive value was the highest for RSV (97.73%) and lowest for Flu A (61.54%); negative predictive values were ≥98.00% for all respiratory viruses. LOAA recorded an “almost perfect” agreement (κ ≥0.88) with rRT-PCR for RSV, SARS-CoV-2, and Flu B and good agreement for Flu A (κ = 0.72). LOAA is sensitive in detecting SARS-CoV-2, RSV, and Flu B infections; however, minor improvements for Flu A are required.

**IMPORTANCE:**

This study presents the potential of a digital PCR as a highly sensitive and reproducible tool for detecting respiratory viruses in Ghana, where robust diagnostic methods are essential for managing public health challenges. By evaluating the novel Lab-On-An-Array (LOAA) system, we provide its critical operational performance against the gold-standard rRT-PCR for detecting severe acute respiratory syndrome coronavirus 2, respiratory syncytial virus, and influenza viruses. Our findings show that LOAA demonstrates excellent agreement with rRT-PCR for most viruses, offering a promising alternative for respiratory virus surveillance and diagnosis. This research is particularly significant for resource-limited settings, as it supports the adoption of advanced molecular diagnostics to improve early detection and response to respiratory infections. Minor refinements for specific viruses, such as influenza A, could further enhance its utility in clinical and epidemiological applications.

## INTRODUCTION

Respiratory viruses include a wide range of viruses that infect cells of the respiratory tract and are associated with significant morbidity and mortality worldwide. Respiratory viruses are responsible for about 90% of upper respiratory tract infections and 30% of lower respiratory tract infections (1). Common human respiratory viruses include influenza viruses, respiratory syncytial virus, adenoviruses, rhinoviruses, and coronaviruses. Respiratory viruses belong to diverse families and differ in their mode of transmission, seasonality of circulation, and disease severity (2). Since 2019, severe acute respiratory syndrome coronavirus 2 (SARS-CoV-2) has emerged as an important respiratory virus accounting for over 776 million confirmed cases and 7.1 million deaths worldwide as of 16 July 2024 (3).

To contain and minimize the spread of infection, prompt and accurate diagnosis is of utmost importance to detect, isolate, treat, and trace infected individuals. Clinical diagnosis of SARS-CoV-2 infection is complicated by nonspecific symptoms such as fever, chills, cough, headache, shortness of breath, and sore throat that may be caused by other respiratory viruses or bacteria (4). The similarities in symptoms pose a challenge to clinicians; hence, the need for laboratory diagnosis to differentiate between infections caused by respiratory viruses, bacteria, and fungi.

Conventional viral diagnostic methods, such as viral culture, immunofluorescence assays, and serological techniques, are time-consuming, labor intensive, and more prone to cross-reactivity relative to emerging methods such as real-time reverse transcription PCR (rRT-PCR). Rapid diagnostic tests, such as lateral flow assays, are relatively inexpensive and easy to use; however, they are often of limited value in diagnostics due to poor sensitivity and specificity (5). Currently, the World Health Organization has established rRT-PCR, a nucleic acid amplification test, as the gold standard to detect SARS-CoV-2 (6). rRT-PCR makes use of target specific primers and probes to accurately distinguish between viral pathogens, thus conferring higher sensitivity and specificity to these types of assays. In addition, rRT-PCR is capable of high throughput screening and can provide an estimation of viral load by the use of cycle threshold (Ct) values with an appropriate standard or calibration curves, although it is important to avoid sole reliance on Ct values when estimating viral load, given that these values can be affected by other factors including inherent inter-run variability (7).

To compensate for the limitations of rRT-PCR for diagnosis, digital PCR (dPCR) assays are becoming increasingly popular for use in both research and diagnostic laboratory settings (8, 9). The dPCR method is similar to rRT-PCR in that specific primers and probes bind to complementary segments in the target organism genome (10). However, dPCR differs from rRT-PCR in that each reaction is divided into thousands of individual partitions with some partitions containing the target molecule while others are lacking. After thermal cycling, each partition is then analyzed for the presence or absence of a fluorescent signal. The ratio of partitions with a fluorescent signal to the total number of partitions is analyzed using Poisson distribution to calculate the copy number of the target molecule. As such, dPCR assays do not require standard curves for quantification and provide precise and comparatively more reproducible results than rRT-PCR (9). Unlike rRT-PCR, partitioning in dPCR ensures that thousands of reactions occur simultaneously, thus increasing tolerance to inhibitors and sensitivity to detect low copy numbers of target molecules (11, 12).

In this study, we evaluated the clinical performance of a Lab-On-An-Array (LOAA) digital real-time PCR analyzer (Optolane Technologies Inc, South Korea), a novel platform for the multiplex detection of four respiratory viruses—SARS-CoV-2, RSV, Flu A, and Flu B—using rRT-PCR as the gold standard. To date, the evaluation of LOAA has been limited to singleplex, small-scale laboratory-based studies using cultured cells (8) and already confirmed samples for analysis (10). In a previous study, LOAA perfectly matched rRT-PCR (Allplex 2019-nCoV Kit, Seegene Inc, Korea) for singleplex detection of COVID-19 (10). LOAA was reported to be 10-fold more sensitive than rRT-PCR; however, the results of this study may not be generalizable due to sampling bias, small sample size, and poor study design adopted for the study (10).

The current study is the first to evaluate the performance of LOAA using prospective clinical samples collected from the field in Ghana. The sensitivity, specificity, positive predictive value (PPV), negative predictive value (NPV), area under curve (AUC), kappa, and accuracy of LOAA for differential diagnosis of SARS-CoV-2, RSV, Flu A, and Flu B using rRT-PCR as the gold standard were assessed.

## MATERIALS AND METHODS

### Study area

**Fig 1:**
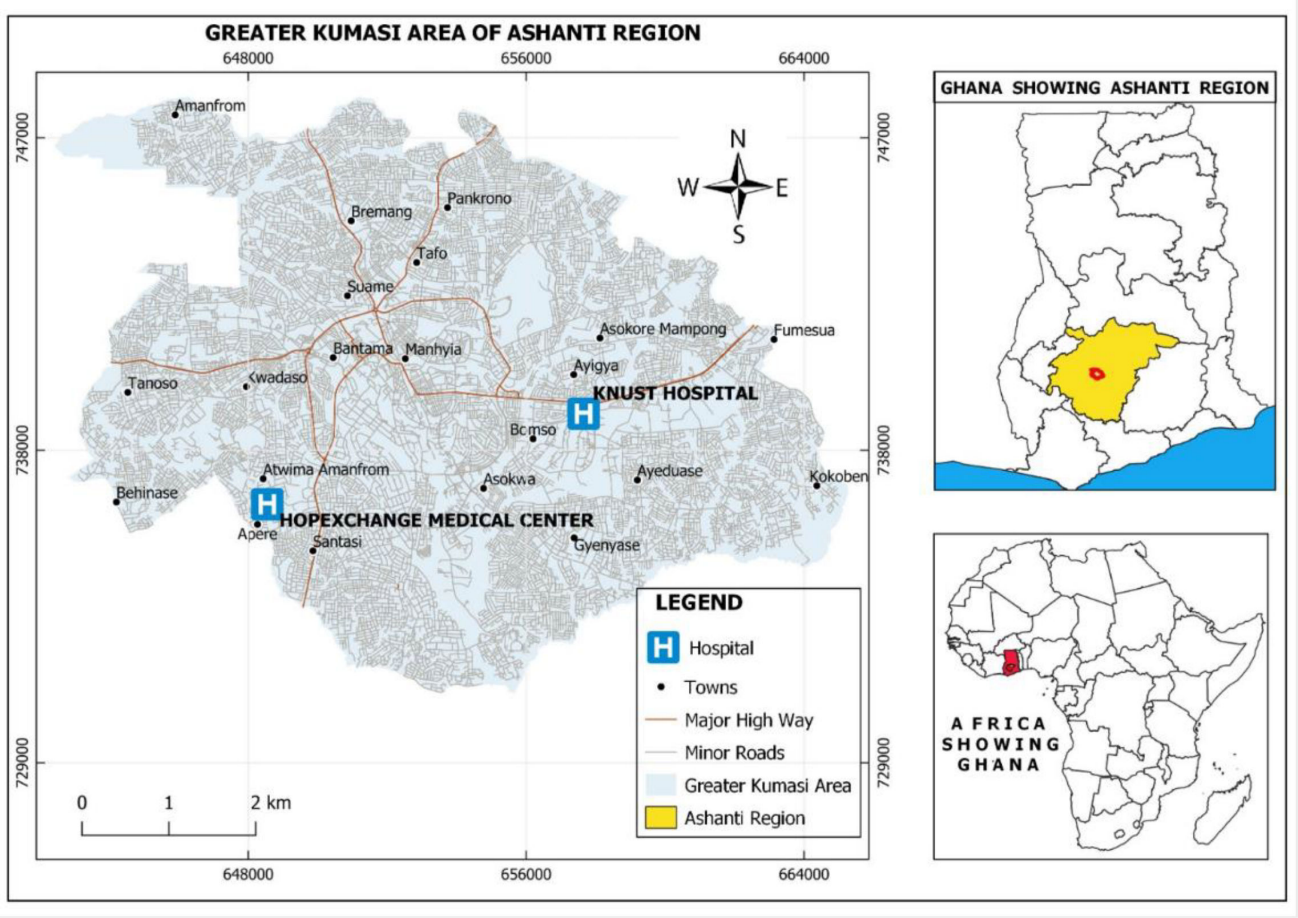
Map of the Ashanti Region showing the study sites. The map was produced using ESRI ArcGIS 10.8, with base layers sourced from https://www.openstreetmap.org. Regional boundary shapefiles were obtained from https://gadm.org/download_country_v4.html and used in accordance with their licensing terms.

**Fig 2:**
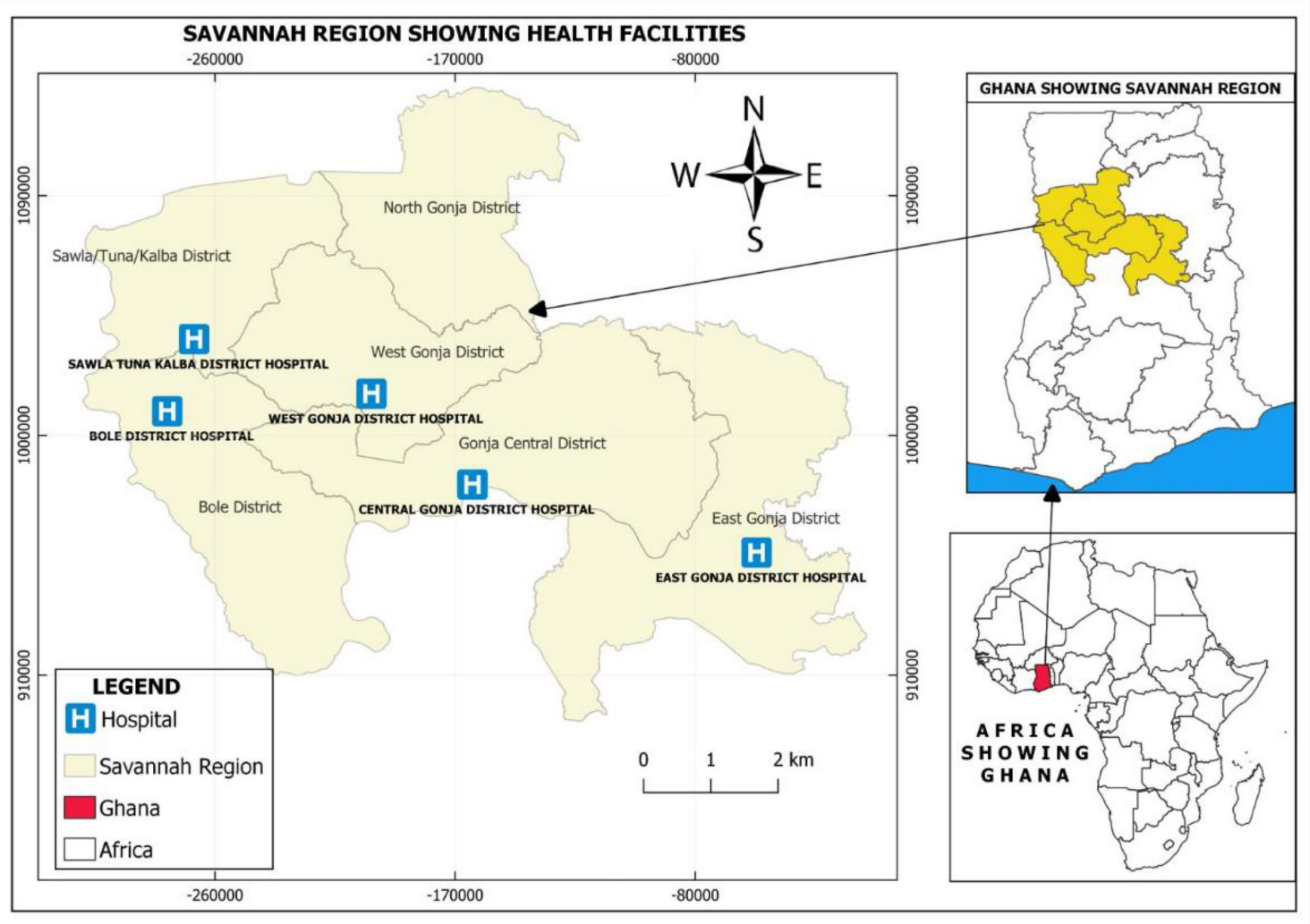
Map showing the location of the study sites in the Savannah Region of Ghana. The map was produced using ESRI ArcGIS 10.8, with base layers sourced from https://www.openstreetmap.org. Regional boundary shapefiles were obtained from https://gadm.org/download_country_v4.html and used in accordance with their licensing terms.

The study was conducted at seven health facilities located within the Ashanti and Savannah Regions of Ghana. In the Ashanti Region, two health care facilities—KNUST Hospital and HopeXchange Medical Center (Fig. 1) and in Savannah Region, five health care facilities—Bole, Sawla-Tuna-Kalba, West Gonja, Central Gonja, and East Gonja District Hospitals (Fig. 2) were selected for the study.

These two regions were purposively selected for the study based on anecdotal knowledge of respiratory viral diseases in the health facilities located in these regions. Located in the southern part of Ghana, the Ashanti Region doubles as the second largest region in terms of population size and the region with the second highest number of confirmed COVID-19 cases (13). About 61% of its inhabitants live in urban areas (14). The Savannah Region occupies the northern part of Ghana and is the largest region in terms of land mass. Notwithstanding, the region is the second least populated with about 70% of its inhabitants living in rural areas (14). As of 7 April 2024, the Savannah Region had the least number of COVID-19 cases in Ghana (13).

### Study design and study population

This was a cross-sectional hospital-based study conducted between August 2022 and January 2023. The inclusion criterion for enrollment of participants in the study was a clinical presentation with a suspected case of respiratory tract infection as defined by two or more of the following symptoms: fever, cough, headache, loss of smell, shortness of breath, nausea, vomiting, general weakness, loss of appetite, muscle pain, and diarrhea. Participants of any age or sex were eligible for enrollment in the study. The aim of the study was explained to all participants in their local language, and their consent was obtained before enrollment. Written informed consent was obtained from participants who were 18 years of age and above, while parental/guardian consent and child assent were obtained for those below 18 years of age. Study participants who could not provide informed consent were excluded from the study.

### Sample size determination

The sample size was pre-calculated as the minimum number of samples required to attain 95% sensitivity and specificity assuming a 5% margin of error at a 95% confidence level (α  =  0.05, power 1 – β  =  0.20) using previously described formulas by Hajian-Tilaki (15). To calculate the final sample size, a previous prevalence of 43.2% was used (16). After substituting in the variables, a minimum sample size of 299 was required for the study.

### Collection of clinical specimens

Oropharyngeal specimens were collected from the participants by trained laboratory personnel using flocked sterile swabs. Swabs were taken at the posterior oropharynx area without touching the tongue, as previously described (17). Afterward, the swabs were kept in well-labeled tubes and transported in 1 mL viral medium on ice to the Genomic and Infectious Disease Laboratory at Kwame Nkrumah University of Science and Technology, Kumasi, Ghana, for further testing.

### Laboratory procedures

#### Experimental controls

Prior to testing clinical specimens, experimental controls were obtained from Kumasi Centre for Collaborative Research in Tropical Medicine, Kumasi, Ghana, to validate assay protocols for the detection of respiratory viruses by LOAA and rRT-PCR. The controls included archived SARS-CoV-2, RSV, Flu A (H1N1, H3N2), and Flu B samples confirmed positive by rRT-PCR.

#### Extraction of viral RNA

Nucleic acid extraction was performed at ambient temperature for the control and clinical samples in a class II biosafety cabinet. Viral RNA was extracted using QIAamp Viral RNA Mini Kit (Qiagen Diagnostics GmbH, Germany) following the manufacturer’s instructions. Viral RNA was eluted into 60 µL buffer AVE and stored at −20°C prior to molecular diagnosis.

#### Real-time RT-PCR

Real-time RT-PCR amplification was performed on the CFX96 Touch Real-Time PCR Detection System (Bio-Rad Inc, USA) for the detection of four respiratory viruses (Flu A, Flu B, SARS-CoV-2, and RSV). The multiplex FluoroType SARS-CoV-2/Flu/RSV Ver 1.0 Kit (Hain Lifescience GmbH, Germany) was used to qualitatively detect respiratory viruses following target specific protocols and primers according to the manufacturer’s instructions. The kit targets the RNA-dependent RNA polymerase (RdRP) and nucleocapsid (N) genes for SARS-CoV-2, the matrix protein 1 (M1) sequence for influenza A, the hemagglutinin sequence for influenza B, and the nucleoprotein (N) sequence for RSV. Real-time RT-PCR was carried out in a 20 µL reaction volume consisting of 8 µL master mix and 12 µL of RNA template. Respective volumes of reagents were adjusted based on final concentration for each primer and probe as specified in the manufacturer’s instruction. The cycling conditions consisted of initial activation at 50°C for 5 min, final activation at 95°C for 20 seconds, denaturation at 95°C for 3 seconds, and annealing/extension at 60°C for 26 seconds. After 50 cycles, the reactions were incubated at 4°C for 1 min. Samples with Ct values of 40 and below were interpreted as positive, and those greater than 40 or not detected were interpreted as negative according to a modified manufacturer’s instructions.

#### LOAA

**Fig 3:**
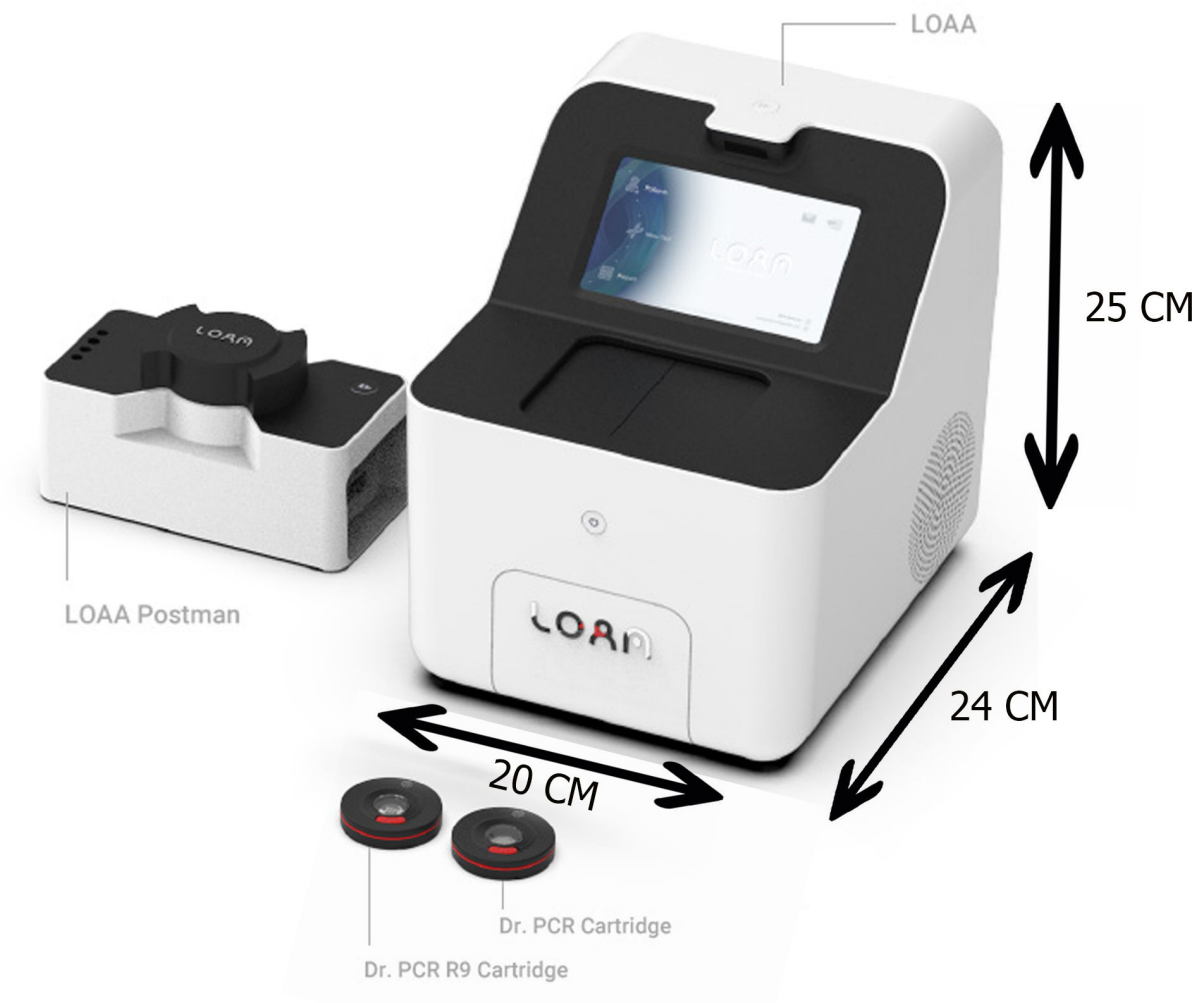
LOAA Analyzer.

The LOAA dPCR has a compact size, measuring 24 cm in length, 20 cm in width, and 25 cm in height. The Genoplexor COVID-19/Flu/RSV Detection Kit (Optolane Technologies Inc, South Korea) with a dynamic range of 5–7 log was used for real-time qualitative detection of SARS-CoV-2 (RdRp/E gene), Flu A (M gene), Flu B (NP gene), and RSV (M gene) RNA using LOAA Analyzer (model: On-Point). A 60 µL reaction mixture consisting of 30 µL of extracted RNA and 30 µL Onestep dRT-PCR (2×) mixture was prepared and loaded onto a chip provided by the manufacturer. The chip was placed in a cartridge and then carefully slotted into the LOAA Analyzer for real-time detection (Fig. 3). The cycling conditions included 52°C of reverse transcription, then denaturation at 97°C, followed by binding of the probe to the specific sequence at 60°C. The reaction mixture was allowed to complete 45 thermal cycles. Samples with Ct values ≤38 were interpreted as positive, while those with Ct values above 38 or not detected were determined to be negative as specified by the manufacturer.

#### Quality control

To ensure reliable diagnosis, internal controls provided by the manufacturers of the kits used in the study, together with the experimental controls obtained, were tested on both LOAA and rRT-PCR prior to testing actual clinical samples. Internal controls were included alongside every batch of clinical samples tested by LOAA and rRT-PCR to validate the results. Biosafety techniques and standard operating procedures were strictly adhered to during processing. Testing was carried out by trained laboratory professionals in a clean working environment.

#### Data analysis

**Fig 4:**
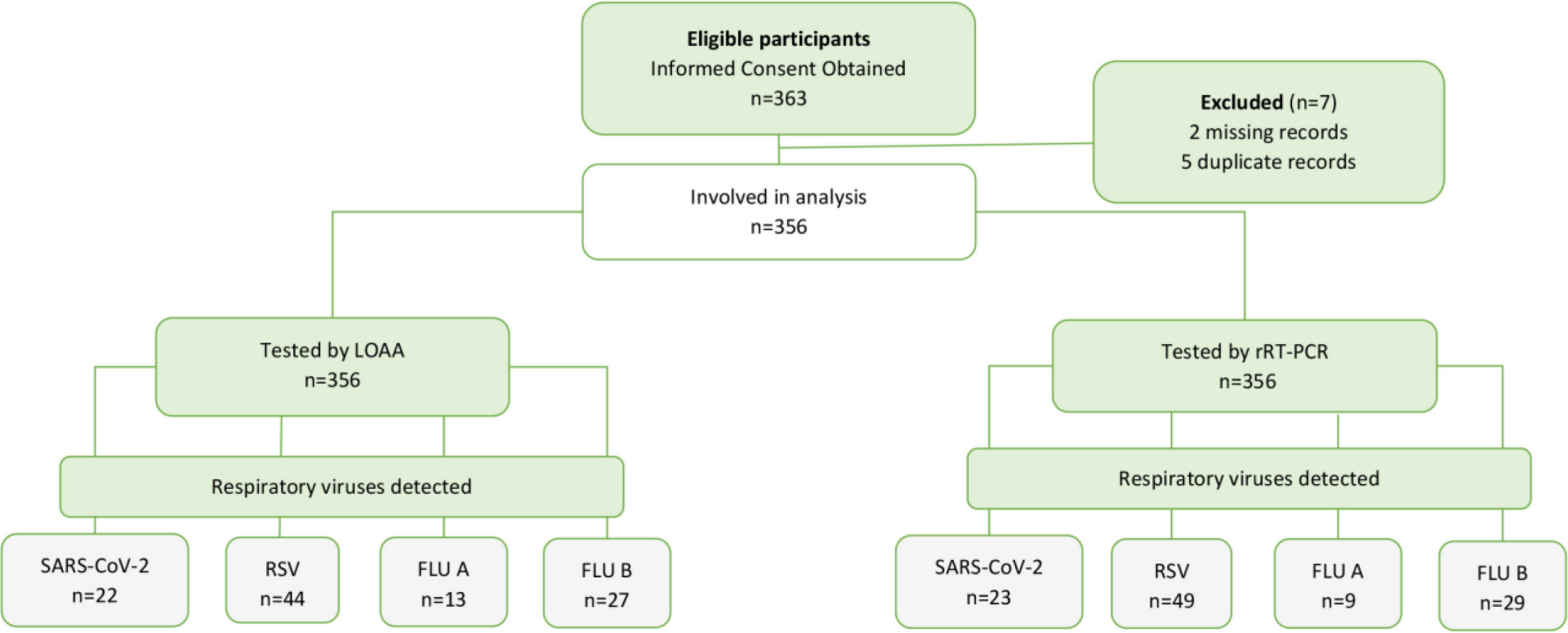
Flowchart describing participant recruitment and tests performed.

Data were entered into Microsoft Excel 2019 (Microsoft Inc, USA) and exported into STATA 14 (College Station, TX, USA) and GraphPad Prism 9.0 (San Diego, CA, USA) for analysis. Incomplete, missing, and duplicate data sets were excluded from the final analysis (Fig. 4). Age and Ct value data were presented as median and interquartile range (IQR), whereas categorical data were presented as proportions. The “diagtest” syntax in STATA was used to calculate sensitivity, specificity, PPV, and NPV. Student’s *t*-test was calculated to compare measurements by LOAA and rRT-PCR. *P*-values <0.05 were considered statistically significant. The AUC for the diagnostic performance of LOAA was obtained from ROC curves, assuming rRT-PCR as a reference point. The kappa value of inter-rater agreement was used to assess the strength of agreement between LOAA and rRT-PCR. Kappa values were interpreted following McHugh’s guidelines; values <0.20 = poor agreement, 0.21–0.40 = fair, 0.41–0.60 = moderate, 0.61–0.80 = good/substantial agreement, and 0.81–1.00 = very good/perfect agreement (18). Spearman’s correlation coefficient, *r*, was used to express the strength of agreement between measurements by LOAA and rRT-PCR. Correlation coefficient, *r*, was interpreted as a “perfect,” “very strong,” “moderate,” “fair,” “poor,” or “no” correlation when *r* = 1.00, 0.80–0.90, 0.60–0.70, 0.30–0.50, 0.10–0.20, or 0.00, respectively (19).

## RESULTS

### Socio-demographic characteristics of the study population

A total of 356 patients were recruited for the study—298 (83.70%) from the Ashanti region and 58 (16.30%) from the Savannah region. All the viruses investigated in the study were detected in each region, with 67.79% (*n* = 202) and 24.14% (*n* = 14) positivity rates in Ashanti and Savannah, respectively. The age of participants ranged from 2 weeks to 83 years, with a median age of 19 years (IQR = 2 weeks–30 years). Detailed socio-demographic characteristics of the study population have been summarized in Table 1.

**TABLE 1:** Characteristics of the study population

Characteristic	Ashanti (*n* = 298) No. (%)	Savannah (*n* = 58) No. (%)	Total (*n* = 356) No. (%)
Gender			
Male	121 (40.60)	20 (34.48)	141 (39.61)
Female	177 (59.40)	38 (65.52)	215 (60.39)
Age group (years)			
≤10	124 (41.61)	0 (0.00)	124 (34.83)
11–20	42 (14.09)	11 (18.97)	53 (14.88)
21–30	73 (24.50)	21 (36.21)	94 (26.40)
31–40	30 (10.06)	7 (12.07)	37 (10.39)
41–50	15 (5.03)	8 (13.79)	23 (6.46)
51–60	5 (1.67)	6 (10.34)	11 (3.09)
61–70	6 (2.01)	3 (5.17)	9 (2.53)
>70	3 (1.00)	2 (3.44)	5 (1.40)
Education			
No formal education	53 (17.79)	8 (13.79)	61 (17.13)
Primary	91 (30.54)	27 (46.55)	118 (33.15)
Secondary	41 (13.76)	12 (20.69)	53 (14.89)
Tertiary	91 (30.54)	0 (0.00)	91 (25.56)
^*a*^No response	22 (7.38)	11 (18.97)	33 (9.27)

^*a*^ No response: This individuals did not provided information on their educational status.

### Experimental controls

A total of 12 experimental controls were obtained for testing all the viruses under study by LOAA and rRT-PCR prior to testing of clinical samples. We used eight positive controls (two per virus) and four negative controls. However, for all experimental controls tested, LOAA qualitatively matched rRT-PCR for the detection of the respiratory viruses (data not shown).

### Prevalence of respiratory viruses by LOAA and rRT-PCR

Of 356 clinical specimens tested, rRT-PCR detected 110 (30.90%) respiratory viruses while LOAA detected 106 (29.78%). For both rRT-PCR and LOAA, RSV was the predominant respiratory virus, followed by Flu B, SARS-CoV-2, and Flu A. The difference in the detection of respiratory viruses by LOAA and rRT-PCR was not statistically significant (Table 2).

**TABLE 2:** Detection of respiratory viruses by LOAA and rRT-PCR^*a*^

Respiratory virus	rRT-PCR (*n* = 356)	LOAA (*n* = 356)	*P*-value
SARS-CoV-2, *n* (%)	23 (6.46)	22 (6.18)	>0.99
RSV, *n* (%)	49 (13.76)	44 (12.36)	0.66
Flu A, *n* (%)	9 (2.53)	13 (3.65)	0.52
Flu B, *n* (%)	29 (8.15)	27 (7.58)	0.89
*Total, n (%)*	110 (30.90)	106 (29.78)	0.81

^*a*^ Fischer’s exact test was used to compute the *P*-value.

### Clinical performance of LOAA vs rRT-PCR

Using rRT-PCR as a reference, the sensitivity (Sn) of LOAA to detect respiratory viruses ranged between 86.21% (Flu B) and 91.30% (SARS-CoV-2). LOAA showed high specificity (Sp) (>98.00%) for all respiratory viruses tested in the study. LOAA was most sensitive in detecting SARS-CoV-2 (Sn: 91.30%, Sp: 99.70%), while the lowest sensitivity was recorded for Flu B (Sn: 86.21%, Sp: 99.38%). Sensitivity for RSV (Sn: 87.76%, Sp: 99.67%) and Flu A (Sn: 88.89%, Sp: 98.54%) was slightly higher, though specificity remained consistently high across all viruses. For all respiratory viruses, LOAA showed “very good/almost perfect agreement” (κ = 0.88–0.93) with rRT-PCR except for Flu A (κ = 0.72), which showed “good/substantial agreement” (Table 3). Overall, LOAA produced 9 false positives and 13 false negatives as compared to the gold standard rRT-PCR assay for all respiratory viruses tested.

**TABLE 3:** Clinical performance of LOAA vs rRT-PCR

Respiratory virus	LOAA	rRT-PCR			Sensitivity (95% CI)	Specificity (95% CI)	PPV (95% CI)	NPV (95% CI)	AUC	Accuracy %	Kappa
	Result	Positive	Negative	Total							
SARS-CoV-2	Positive	21	1	22	91.30 (73.20–98.45)	99.70 (98.32–99.98)	95.45 (78.20–99.77)	99.40 (97.84–99.89)	0.75	99.16	0.93
	Negative	2	332	334							
	Total	23	333	356							
RSV	Positive	43	1	44	87.76 (75.76–94.27)	99.67 (98.18–99.98)	97.73 (88.19–99.88)	98.08 (95.87–99.12)	0.59	98.03	0.91
	Negative	6	306	312							
	Total	49	307	356							
Flu A	Positive	8	5	12	88.89 (56.50–99.43)	98.54 (96.62–99.37)	61.54 (35.52–82.29)	99.70 (98.34–99.98)	0.78	98.31	0.72
	Negative	1	342	343							
	Total	9	347	356							
Flu B	Positive	25	2	27	86.21 (69.44–94.50)	99.38 (97.76–99.89)	92.59 (76.63–98.63)	98.77 (96.87–99.52)	0.70	98.31	0.88
	Negative	4	325	329							
	Total	29	327	356							

### Ct values of respiratory viruses by LOAA and rRT-PCR

**Fig 5:**
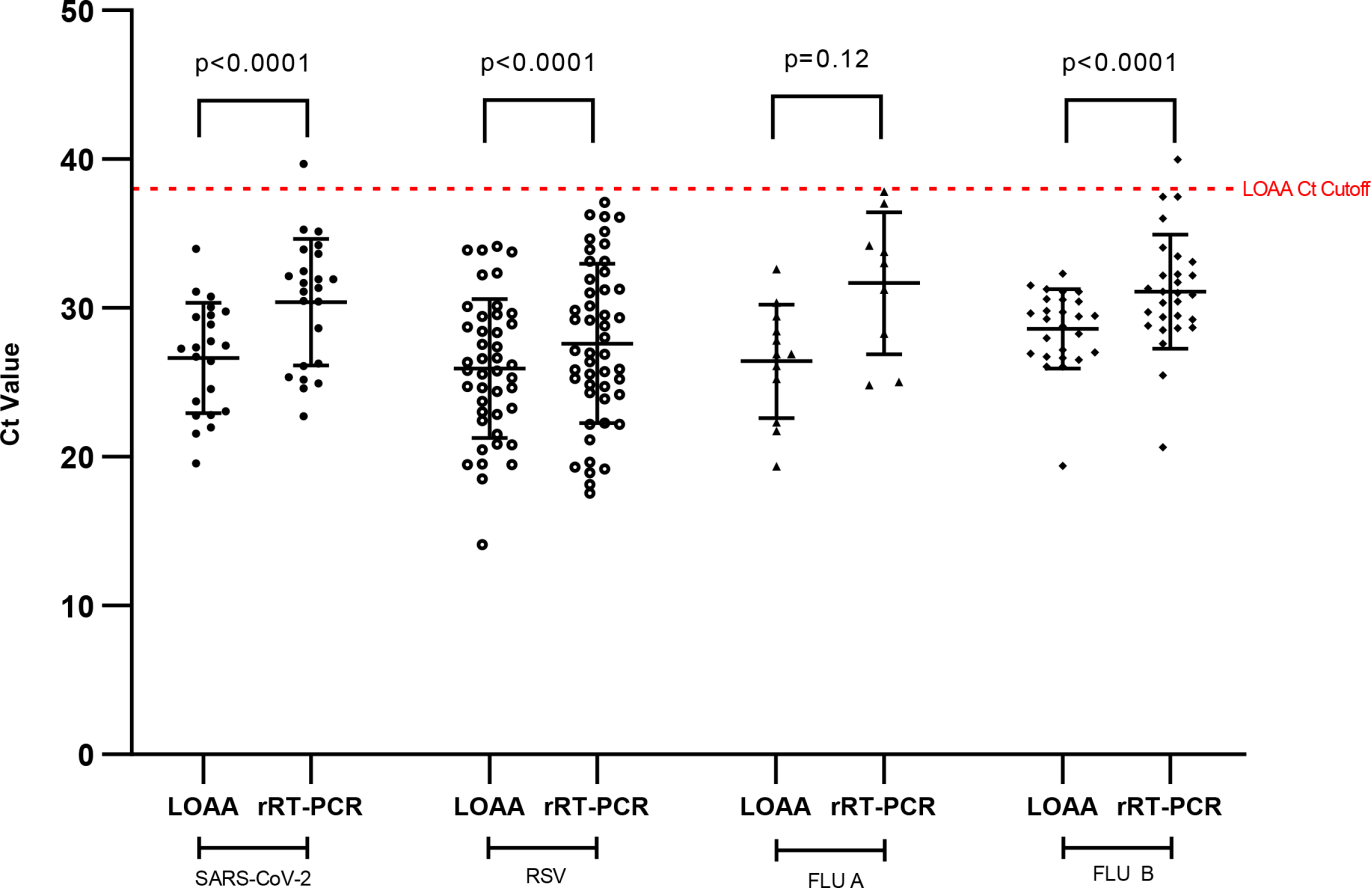
Comparing Ct values of individual respiratory viruses detected and assay sensitivity of rRT-PCR and LOAA. The red dotted line indicates the Ct cutoff value of LOAA. Pearson’s correlation was used to compute the *P*-value.

The Ct values of respiratory viruses detected by rRT-PCR and LOAA ranged from 17.56 to 39.97 and 14.12 to 34.14, respectively. The median Ct value, 29.79 (IQR: 25.61, 32.89), detected by rRT-PCR was significantly higher (*P* < 0.0001) than that of LOAA, 27.25 (IQR: 24.45, 29.62) (Fig. 5).

### Correlation of LOAA and rRT-PCR Ct values for detection of respiratory viruses

**Fig 6:**
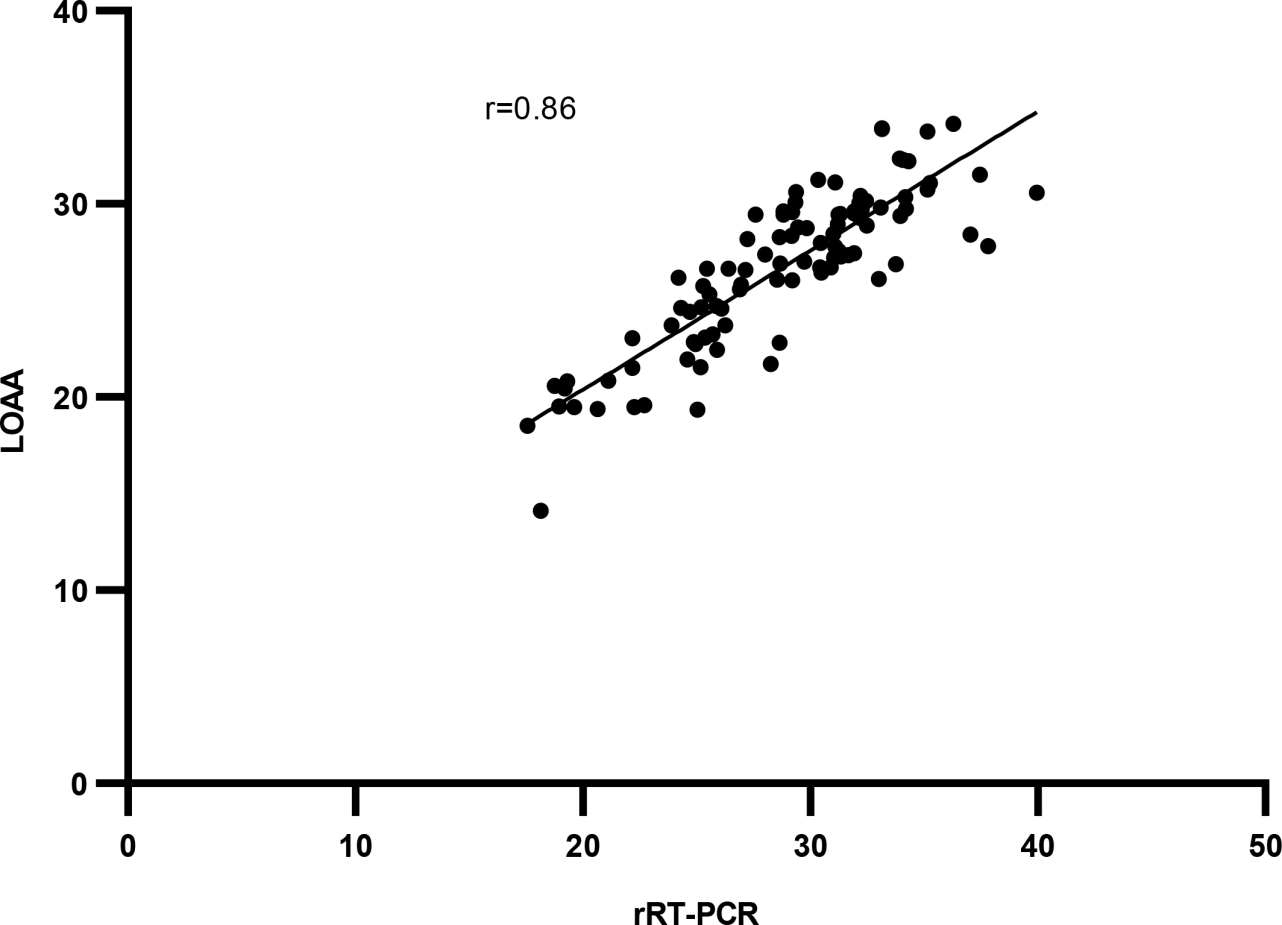
Correlation of Ct values detected by LOAA and rRT-PCR.

**Fig 7:**
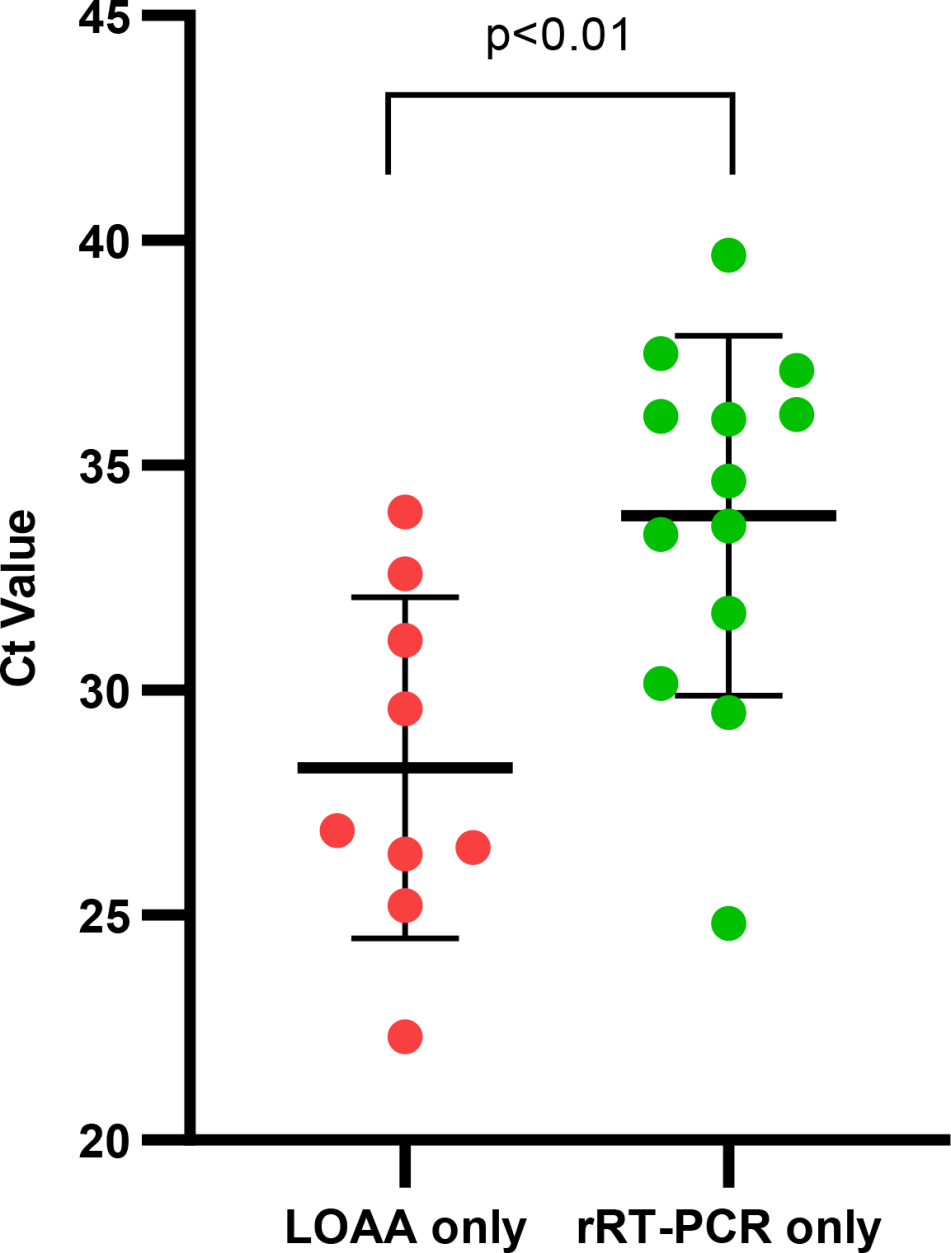
Ct of infections detected by rRT-PCR and LOAA only.

Spearman’s correlation coefficient, *r*, was calculated to determine the strength of the agreement between Ct values of LOAA and rRT-PCR. All individual respiratory viruses showed significant differences in Ct values for LOAA and rRT-PCR except for Flu A, which had comparable Ct values for LOAA and rRT-PCR (Fig. 5). For the detection of respiratory viruses, a “very strong” positive correlation, rs = 0.86 (95% CI: 0.79, 0.90), was observed between the combined Ct values by LOAA and rRT-PCR as shown in Fig. 6. For the detection of respiratory viruses by either method only, an unpaired *t*-test was used to compare the mean Ct values, which was found to be significant (Fig. 7).

### Comparison of respiratory viral coinfections detected by LOAA and rRT-PCR

Coinfections were identified in the study, with rRT-PCR detecting four cases and LOAA identifying three cases. Both methods detected one case of SARS-CoV-2/RSV coinfection. LOAA uniquely detected SARS-CoV-2/Flu B and Flu A/Flu B, which were missed by rRT-PCR, while rRT-PCR identified three cases of RSV/Flu B that were not detected by LOAA (Table 4).

**TABLE 4:** Respiratory viral coinfection detected by LOAA and rRT-PCR

Coinfection	rRT-PCR, No.	LOAA, No.
SARS-CoV-2/RSV	1	1
SARS-CoV-2/Flu B	0	1
Flu A/Flu B	0	1
RSV/Flu B	3	0
Total	4	3

### Discordant results

A total of 22 discordant test results were recorded in the study. Out of this number, 13/22 (59.09%) infections were exclusively detected by rRT-PCR, while 9/22 (40.90%) were detected by LOAA only.

## DISCUSSION

Prompt and accurate detection of infectious pathogens, particularly epidemic/pandemic-prone viruses, is critical for successful disease management and control. During the height of the COVID-19 pandemic, manufacturers of biomedical diagnostic devices were faced with the task of developing highly sensitive and rapid tests to support testing and tracing of suspected infected individuals. Gold-standard rRT-PCR, despite its sensitivity, is time-consuming and requires skilled labor (20, 21), whereas most rapid diagnostic tests generally lacked the needed sensitivity and specificity for active case detection (20, 22). It was during this period that Optolane Technologies Inc (Seongnam, South Korea) launched LOAA, a third-generation PCR and a novel compact-sized digital PCR equipment capable of diagnostic applications.

LOAA is a separation-type dPCR instrument that makes use of semiconductor chip-based microelectromechanical system technology to significantly reduce the size of the equipment. LOAA is advantageous over other currently commercialized platforms owing to its compact size, rapid turnaround time, precise measurement, and potential for improved clinical utility (9, 23). Early versions of LOAA were designed to solely detect SARS-CoV-2 (Dr. PCR 20K COVID-19 Detection Kit) and have been reported to show absolute sensitivity and perfect diagnostic agreement with commercially available rRT-PCR-based tests performed on Bio-Rad’s CFX96 (10) and QX200 droplet dPCR platforms (8). Recently, LOAA has been equipped for multiplex detection of common respiratory viruses by the aid of the Genoplexor COVID-19/RSV/Flu Detection Kit (Optolane Technologies Inc, South Korea). In this study, we assessed the clinical performance of LOAA for multiplex detection of SARS-CoV-2, Flu A, Flu B, and RSV using a well-established rRT-PCR assay as the reference standard.

Compared to rRT-PCR, LOAA showed sensitivities of 91.30%, 87.76%, 88.89%, and 86.21% for the detection of SARS-CoV-2, RSV, Flu A, and Flu B, respectively. The sensitivity of LOAA to detect SARS-CoV-2 (91.30%) in the present study was lower than in previous studies in Korea (8, 10, 24). This observation may be explained by three main factors: (i) the current study reports clinical sensitivity based on field evaluations, whereas those cited were laboratory-based studies using already confirmed archived samples, (ii) sensitivity in the current study was estimated from multiplex reaction, while the previous studies were obtained from singleplex assays, and (iii) the present study focuses on oropharyngeal specimens, while the earlier studies included a variety of specimen types in their analyses. The variability in field and laboratory conditions, differences in study design, sample size, and specimen type among the study participants in the current and previous studies may have accounted for the differences in LOAA sensitivity to detect SARS-CoV-2 (25, 26). There are conflicting reports regarding the sensitivity of multiplex and singleplex assays to detect respiratory viruses by nucleic acid amplification tests. While several studies (27–30) showed comparable detection by both assays, other authors (31–34) reported slightly higher sensitivity of singleplex assays than multiplex assays, particularly at the detection of low levels of viremia. The relatively low sensitivity of multiplex assays has been attributed to the competition among different targets for primers and other reagents, which may lead to low amplification efficiency (35, 36). Notwithstanding, multiplex assays are ideally more preferred in the laboratory due to their ability to simultaneously detect multiple infections, thus reducing turnaround time, preserving staff resources, and conserving expensive reagents (30). Despite the difference in sensitivity, the specificity and kappa of LOAA to detect SARS-CoV-2 were similar to previous studies (8, 10, 24).

For both tests, RSV was the predominant respiratory virus detected in the study. This finding was not particularly surprising since the majority of the study participants were aged 10 years and below (Table 1). It has been established that RSV is a common cause of acute respiratory infection among children under 5 years of age (37). Few multiplex dPCR assays have been developed to detect RSV and other respiratory viruses, with very limited data about their comparative field evaluations (38, 39). In this study, LOAA recorded the third highest sensitivity for the detection of RSV (87.76%). This sensitivity was higher than those reported by other digital PCR systems (nCounter; RSV-A 74.30%, RSV-B 77.60%) evaluated in Brazil (40). In addition, LOAA reported marginally higher specificity for RSV compared to RSV-A and RSV-B in the Brazilian study (99.67% vs 98.40%, 99.67% vs 97.80%) and accuracy (98.03% vs 95.20%, 98.03% vs 95.30%), respectively. In contrast to the few multiplex dPCR assays for respiratory viruses, there are several multiplex RT-PCR assays developed to target about 2–18 individual respiratory viruses (30, 41–44). Compared to multiplex RT-PCR assays, LOAA sensitivity to detect RSV was lower than reported in earlier studies (33, 41, 45), which reported absolute sensitivity or positive percent agreement with varying reference standards. This may be due to the low Ct threshold of LOAA, hence, its inability to pick up low-density infections. Again, this may be attributed to different primer designs, differences in viral load, optimization, or efficiency of extraction/amplification process between both tests.

From the study, LOAA recorded low sensitivity for the detection of Flu B (86.21%) and Flu A (88.89%). This sensitivity was higher than that of Qiagen ResPlex II, a multiplex RT-PCR assay for the detection of 17 respiratory viruses in a study held in China (32), but was lower than reported in earlier studies (33, 45). The difference in sensitivities may be attributed to differences in patient populations and, most importantly, variability in the reference standards employed in each study (46). Studies by Mark GC et al. revealed a higher sensitivity of ResPlex II when compared to viral culture as a standard but lower sensitivity when compared to singleplex PCR. Despite the relatively low sensitivity, LOAA showed high specificity (98.54%–99.38%) and overall accuracy (98.31%) for the detection of Flu A and B. Together with a PPV/NPV of 92.59%/98.77% and kappa value indicative of “almost perfect agreement” with rRT-PCR, the LOAA remains a viable choice to detect Flu B infections. For Flu A, however, the low PPV (61.54%) but high NPV (99.70%) and kappa value indicative of “good/substantial agreement” suggest LOAA is best used to rule out Flu A infections rather than detecting them.

From the study, LOAA reported a lower percentage of false positive (*n* = 9/22; 40.90%) and a higher rate of false negative (*n* = 13/22; 59.09%) results when compared to rRT-PCR. False positive dPCR results have been attributed to poor assay design and off-target amplification stemming from cross-contamination during experimental setup (47). False negatives, on the other hand, may be attributed to the relatively low detection limit of LOAA, hence, its inability to detect low-density infections. False negative dPCR results at low viral load have been reported in other studies (48, 49).

Overall, our results suggest LOAA is a sensitive and highly accurate platform when compared to the gold standard, rRT-PCR, for the detection of SARS-CoV-2, RSV, and Flu B, with minor improvements needed for Flu A. The ease of operation and portable compact size of LOAA validate the novel platform as an efficient tool for swift diagnostic outcomes, catering to the need for timely clinical decision-making. The LOAA device necessitates a nucleic acid extraction step before testing. Resolving this requirement in the future will enhance the platform’s efficiency, potentially resulting in a significantly improved functionality as a point-of-care device or bedside test device. In the current study, only a few cases of viral coinfection (LOAA = 3/356, rRT-PCR = 4/356) were reported (Table 4). This was lower than previous studies in China (39) where 6/98 viral coinfections were detected by a multiplex digital droplet PCR assay and in Ecuador (50) where 19/158 viral coinfections were detected by multiplex RT-qPCR assays. It is plausible that the variation in the study population, period of the study, and geographical area may have accounted for differences in coinfections in both studies. Our results showed that LOAA perfectly matched rRT-PCR to detect SARS-CoV-2/RSV. It outperformed rRT-PCR in detecting SARS-CoV-2/Flu B and Flu A/Flu B coinfections but failed to detect all three RSV/Flu B coinfections detected by rRT-PCR. Viral coinfections have been implicated in altered viral pathogenicity, disrupted host response, and complicated diagnosis (51). Studies have shown an increase in false negative test results due to masking of target viral RNA by undesired pathogens in coinfections (52). Further studies are recommended to investigate the efficacy of LOAA to detect coinfections—both viral and bacterial-viral—and how it influences its accuracy.

### Conclusion

The emergence of novel highly sensitive digital PCR tests represents a significant breakthrough in overcoming the challenges associated with conventional PCR testing. Our results validate the Genoplexor COVID-19/RSV/Flu Detection Kit performed on LOAA as a highly sensitive tool for the detection of SARS-CoV-2, RSV, and influenza type B (Flu B) with minor improvements required for type A (Flu A). With the omission of the nucleic acid extraction step, LOAA will be more efficient for the detection of SARS-CoV-2 and other respiratory viruses. It goes without saying that the sustainability of this method and its practicality for surveillance testing further outlines its potential for widespread use in monitoring and controlling respiratory virus outbreaks.

### Limitations of the study

One limitation of the study is the potential impact of using oropharyngeal swabs in our study which could have implications on the results. This difference in sampling methods could potentially lead to an overestimation of prevalence, consequently affecting the accuracy of the sample size calculation.

In addition, while LOAA qualitatively matched rRT-PCR for all experimental controls, the small sample size may not provide sufficient assurance for pathogen detection across diverse viral lineages. This limitation constrains the robustness of the validation, and further testing with a broader range of controls is necessary to ensure broader applicability of the results.

Moreover, though the current requirement for standard nucleic acid extraction of LOAA may limit immediate point-of-care use, it ensures high sensitivity and specificity; ongoing efforts to integrate simplified extraction methods are expected to enhance the kit’s accessibility and utility in diverse settings.

Again, the absence of sequencing or other molecular methods to resolve discrepancies between LOAA and rRT-PCR results limits definitive interpretation of diagnostic accuracy.

Finally, the two assays, LOAA and rRT-PCR, use different viral targets, which impact the limits of detection and Ct values. These differences may introduce challenges in making meaningful comparisons between the assays, as they may reflect assay-specific sensitivities rather than true performance equivalency. Future work should aim to harmonize or adjust for these assay variations to improve the validity of cross-comparison.

## Data Availability

The datasetsdata sets supporting the conclusions of this article are included within the article and available online (10.6084/m9.figshare.29606108).
